# A Comprehensive Analysis of Complications in Pediatric Lateral Humeral Condyle Fractures

**DOI:** 10.1007/s43465-025-01404-7

**Published:** 2025-05-20

**Authors:** Miguel Tovar-Bazaga, Luis Moraleda-Novo, Maria Valencia-Mora

**Affiliations:** 1 Department of Orthopaedic and Traumatology Surgery, IIS-Fundación Jiménez Díaz. Av, Reyes Católicos 2. 28040, Madrid, Spain; 2 Department of Orthopaedic and Traumatology Surgery, La Paz University Hospital Pº de La Castellana, 261. 28046, Madrid, Spain

**Keywords:** Children fractures, Humeral condyle, Jakob, Subsequent displacement, Non-union

## Abstract

**Background:**

Complications such as subsequent displacement and non-union in children with non-displaced or minimally displaced lateral humeral condyle fractures (LHCF) may happen. We aim to identify potential prognostic factors that could influence the outcomes of these fractures.

**Material and Methods:**

A retrospective study in a level I trauma center was performed. The patient data included demographics, injured side, fracture displacement, treatment, and the occurrence of subsequent displacement, non-union, or other complications. Fractures were classified according to the Jakob's classification, relying on simple radiographs, and displacement was quantified in millimeters using anteroposterior (AP) and lateral views.

**Results:**

The study sample comprised 89 children (80% Jakob I and 20% Jakob II). The mean age at the time of injury was 6 (3). Treatment was non-surgical in 81% of cases, while 19% received surgery. 24 patients (27%) experienced complications. Correlational analyses revealed that a higher degree of initial displacement showed significant associations with non-union, lateral condyle hypertrophy, and alignment disturbance. Predictive performance analysis indicated that displacement exceeding 1.5 mm should be surgically treated.

**Conclusions:**

Following non-displaced or minimally displaced LHCF, our study revealed an 8% occurrence of subsequent displacement and a 2% rate of non-union. An association between the initial degree of fracture displacement and the occurrence of non-union, lateral condyle hypertrophy, and alignment disturbances was observed. Fractures exhibiting displacement greater than 1.5 mm might be deemed appropriate for surgical intervention.

**Level of Evidence:**

IV.

## Introduction

**Fig. 1:**
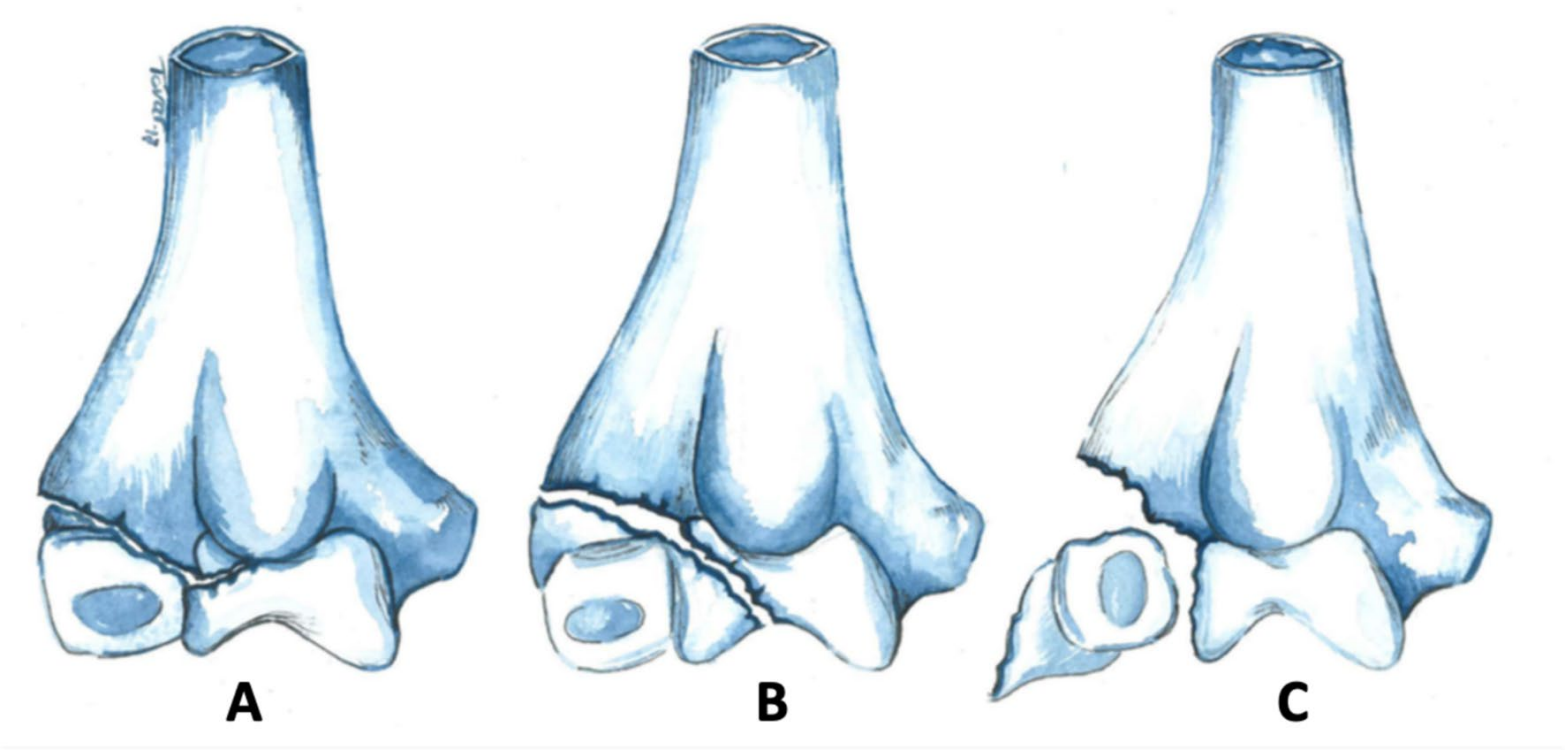
Lateral Humeral Condyle Fracture Jakob’s classification. **A** Jakob type I: nondisplaced. **B** Jakob type II: minimally displaced < 2 mm. **C** Jakob type III: displaced ≥ 2 mm and/or rotated. Original image

Lateral humeral condyle fractures (LHCF) represent the second most prevalent type of elbow fracture in children [1]. While Milch's classification is widely cited, it lacks practical utility in establishing a treatment algorithm [2]. In contrast, Jakob's classification proves beneficial in guiding treatment decisions [3] (Fig. 1). Surgical intervention is clearly indicated for Jakob's type III fractures. However, there remains ongoing controversy regarding the preferred treatment for Jakob's types I and II fractures [4]. Song’s classification has emerged as a new but complex classification of LHCF [5].

Some authors consider all Jakob’s types of fractures unstable due to the forces exerted by the collateral radial ligament and extensor muscles and propose to treat them surgically from the beginning [6]. However, surgically treating fractures that would never displace or fail to heal would mean to take unnecessary risks [7].

On the contrary, some authors suggest non-operative treatment based on a stabilizing cartilaginous hinge that would prevent subsequent displacement, but with the necessity of an arthrogram [8, 9]. Other authors advocate initial surgical management to avoid this potential secondary displacement and eventually non-union which would lead to a more aggressive delayed surgical plan [10]. Secondary displacement has been described in 15% of minimally displaced fractures [11]; while 14.5% of non-displaced or minimally displaced fractures presented nonunion when non-operatively treated [12, 13]. Other complications non-specific of this fracture, such as avascular necrosis (AVN) with a fishtail deformity, may occur. These complications highlight the clinical implications of mismanagement and the need for clear guidelines in treating these fractures.

The aims of the study were: 1) to determine the rate of secondary displacement and non-union associated with non-operative treatment of Jakob’s type I or II fractures; 2) to identify risk factors associated with secondary displacement or nonunion; and 3) to analyze and compare the rates of other complications between non-operative and surgical treatment of Jakob's type I or II fractures.

## Material and Methods

The study was conducted with ethical approval from the Institutional Ethics Review Board (PI-2636). Retrospective data was collected, and patients were identified by reviewing elbow radiographs of children under the age of 15 from January 1990 to January 2021. In total, 717 radiographs were examined.

The treatment decision for each case was made by the attending physician, who chose between splinting or closed reduction and percutaneous pinning (CRPP). Subsequently, all patients received immobilization in a long-arm splint. Within one week after the injury, all patients underwent a follow-up, where anteroposterior (AP) and lateral radiographs were taken. The immobilization was continued for a minimum of 4 weeks with a weekly x-ray control. A standard physical therapy program was not routinely implemented, only contemplated if evolution of the range of motion was presumed incomplete.

The study's inclusion criteria encompassed children below the age of 15, diagnosed with non-displaced or minimally displaced LHCF (Jakob's type I and II). Children with Jakob's type III fractures were excluded due to its initial surgical indication, along with cases involving poor or incomplete radiological exams and insufficient data.

**Fig. 2:**
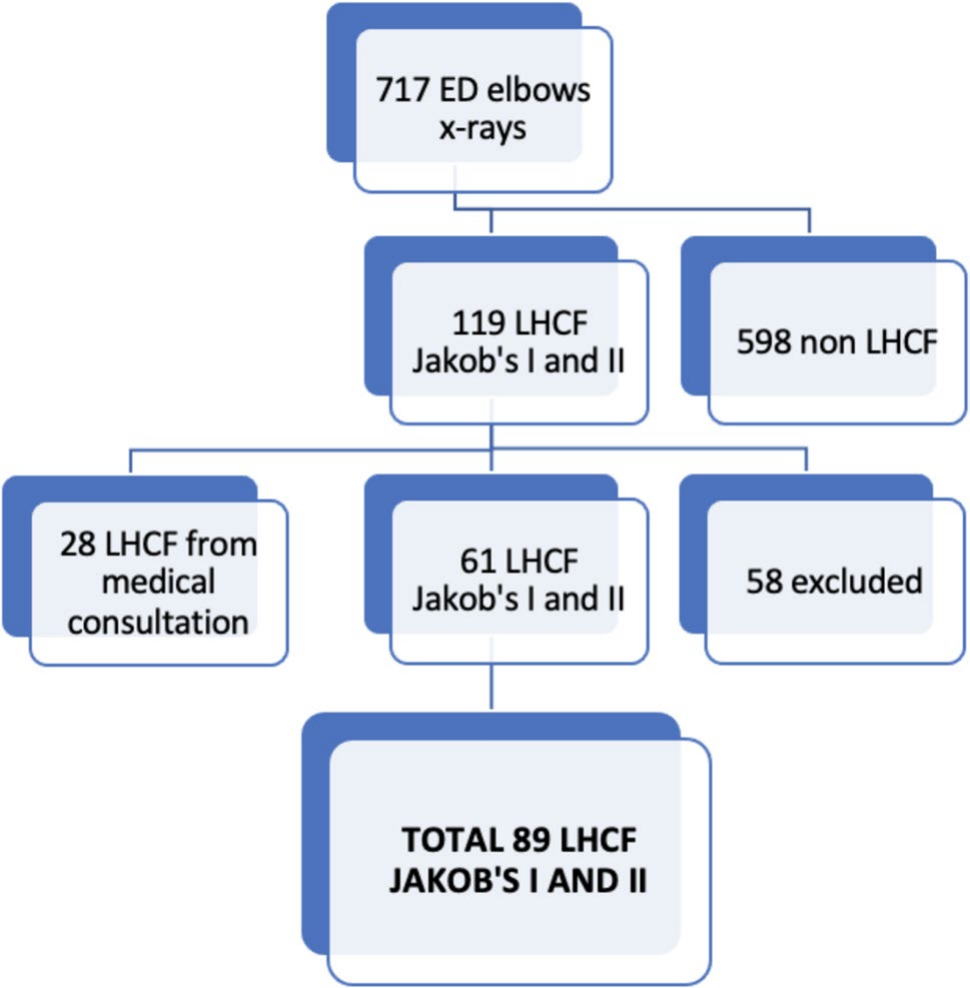
Children population with elbow injuries flowchart

A total of 89 patients met the inclusion and exclusion criteria and were included in the study (Fig. 2). Comprehensive data were collected from the patients' medical records, covering demographic information, laterality, mechanism, duration of immobilization, time to surgery, type of reduction and fixation (if surgery was undertaken), duration of the surgical procedure, any changes in treatment, and complications.

The initial AP and lateral radiographs were used to measure the highest displacement of the fracture in millimeters via PACS software. Fractures were then classified based on Jakob's classification [3]. Subsequent radiographs taken one week after the injury were assessed for any signs of secondary displacement. Non-union was defined as the absence of fracture healing, starting from the eighth week of follow-up [6, 12, 13].

At the latest follow-up, radiographs were thoroughly reviewed to identify any other complications, such as lateral humeral condyle hypertrophy, avascular necrosis (fish-tail deformity or sclerosis with delayed healing), or alignment disturbances (cubitus varus/valgus). To assess the alignment of the elbow, measurements were taken for Baumann's angle, radiologic carrying angle, and lateral humeral condylar angle. Complications were compared between fractures surgically treated and those non-operatively.

The Mann–Whitney *U* test was used for the analysis of quantitative variables. Qualitative variables were studied by the Chi square test or the Fisher’s exact test. A significant difference was defined as p < 0.05. A receiver operating characteristics (ROC) curves searching for predictive measures were made.

## Results

Our study includes 89 children with an average age at injury of 6 ± 3 years old, and a mean follow up of 9.4 months (median 3). There were 71 (80%) Jakob’s type I fractures and 18 (20%) Jakob’s type II fractures. X-rays views were consistent in all the patients. Mean displacement of the fracture was 1.02 mm (0–4; median 1) in the AP radiograph, and 1.42 mm (0–6; median 1) in the lateral. Demographic data is shown in Table 1. The occurrence of a Jakob’s type I or type II fracture was not related to age at the time of injury (p = 0.74), gender (p = 0.59) or laterality (p = 0.6). However, Jakob’s type I fractures were statistically related to a low-energy traumatism (p = 0.03).

**Table 1:** Demographics

Variable	Results
Number of patients	89
Gender (Female/Male)	56/33
Age (y)	6 ± 3
Laterality (Right/Left)	36/53
Injury mechanism (LowE/HighE)	73/16
Jakob classification (I/II)	71/18
Initial AP displacement (mm)	1.02 (SD 1.22 median 1)
Initial lateral displacement (mm)	1.42 (SD 1.6 median 1)
Initial treatment (conservative/surgery)	72/17
Reduction (Open/Close)	7/10
Fixation (Wire/screw)	16/1
First appointment (d)	9.8 (SD 2.3 median 11)
Immobilization time (d)	33.4 (SD 11.6)
Consolidation time (d)	43.4 (SD 18.7)
Follow-up (m)	9.4 (SD12.7)

Regarding treatment, 81% of the fractures were non-operatively treated. Majority of Jakob’s type I (97%) was non-operatively treated, while majority of Jakob’s type II (78%) was surgically treated (p < 0.001) (Table 2). We performed an arthrogram in 8 patients followed by CRPP, after an unsuccessful attempt of closed reduction. Open reduction and internal fixation (ORIF) was performed if a closed reduction was not possible even with the arthrogram’s aid. Only one patient required an ORIF with a single screw, the rest of them (16) K-wire open pinning was sufficient. We did not find a statistical relationship between initial treatment and age (p = 0.11), gender (p = 0.11), laterality (p = 0.79) or injury’s mechanism (p = 0.145). The mean period of immobilization was 33 days (SD 11.6), higher in Jakob’s type II (p < 0.01) and surgical fractures (p = 0.03).

**Table 2:** Results (bold type: statically significant)

Variable	Results (p)
Surgical treatment (n, Jakob I/II)	2/14 **(< 0.01)**
Radiological follow-up (m)	
Jakob I/II	4/20 **(< 0.01)**
Conservative/Surgical	6/23 **(< 0.01)**
Time of immobilization (d)	
Jakob I/II	31/41 **(< 0.01)**
Conservative/Surgical	32/37 **(0.03)**
Time to consolidation (d)	
Jakob I/II	47/53 (0.13)
Conservative/Surgical	49/54 (0.57)
Lateral humeral condylar angle (grades)	
Jakob I/II	35/38 (0.07)
Conservative/Surgical	34/39 **(0.05)**
Carrying angle (grades)	
Jakob I/II	8/12 **(0.05)**
Conservative/Surgical	10/11 (0.25)

Secondary displacement occurred in 7 patients (4 Jakob’s type I and 3 Jakob’s type II), all of them initially non-operatively treated. The rate of secondary displacement among non-displaced or minimally displaced LHCF non-operatively treated was 7.8%. We were unable to find a statistical relation between secondary displacement and initial treatment, fracture type, and initial displacement in the AP or lateral views (Table 3). All cases with secondary displacement were surgically treated (ORIF with k-wire fixation in six, and closed reduction and k-wire fixation in one).

**Table 3:** Results (bold type: statically significant)

Variables		Results (p)
Consolidation	Jakob I/II	**0.05**
Yes 87/89	Conservative/Surgical	0.34
No 2/89	AP initial X-ray	0.07*
	Lateral Initial X-ray	**0.02**
Secondary displacement	Jakob I/II	0.15
Yes 7/89	Conservative/Surgical	0.34
No 82/89	AP initial X-ray	0.31
	Lateral initial X-ray	0.43
Lateral condyle hypertrophy	Jakob I/II	0.36
Yes 72/89	Conservative/Surgical	**0.05**
No 17/89	AP initial X-ray	**0.01**
	Lateral initial X-ray	**0.03**
Alignment disturbances	Jakob I/II	0.06*
Yes 6/89	Conservative/Surgical	**0.04**
No 83/89	AP initial X-ray	**0.05**
	Lateral initial X-ray	**0.01**
Avascular Necrosis	Jakob I/II	0.09*
Yes 1/89	Conservative/Surgical	0.12
No 88/89	AP initial X-ray	0.29
	Lateral initial X-ray	0.33
Changes in treatment	Jakob I/II	**0.01**
Yes 8/89	Conservative/Surgical	1
No 81/89	AP initial X-ray	**0.05**
	Lateral initial X-ray	**0.04**

*Statically tendence if p = (0.10–0.05)

The mean time for fracture healing was 43 days (SD 19). There were two cases with a non-union, both Jakob’s type II fractures initially non-operatively treated. Both cases were successfully treated with an ORIF with screws. No subsequent development of cubitus valgus was seen in those patients. The rate of non-union among non-displaced or minimally displaced LHCF non-operatively treated was 2.2%. Non-union was statistically associated with a higher initial displacement in the lateral view. There was a tendency between non-union and displacement in the AP view (Table 3). Predictive ROC curve analysis of initial displacement (mm) in the AP radiograph showed an area of 0.845 (0.721–0.970) for non-union. Our analysis suggests that a displacement greater than 1.5 mm in the AP view has a sensitivity of 100% and specificity of 72% and, in the lateral x-ray, demonstrates a 100% of sensitivity and 64% of specificity both for non-union.

Different complications occurred in 17 patients (19%): 17 hypertrophy of the lateral humeral condyle, 6 mal-alignment (3 cubitus varus, 3 cubitus valgus) and 1 AVN (fish-tail deformity). A hypertrophy of the lateral condyle was the commonest complication (19%), more common in those patients surgically treated (50%) than in those non-operatively (12%) (p = 0.05). It was also significantly higher in females (p < 0.01) and with greater initial displacement both in AP (p < 0.01) and lateral (p = 0.03) views. Alignment disturbances were also associated with surgical treatment (p = 0.04) and greater displacement in the anteroposterior (p = 0.05) and lateral (p = 0.01) views. However, AVN was not related to initial type of treatment (p = 0.12) or displacement in the AP (p = 0.29) or lateral (p = 0.33) views. The patient who suffered an avascular necrosis developed further clinical stiffness and cubitus varus and underwent an open arthrolysis and valgus osteotomy.

Changes in initial treatment from non-operatively to operatively occurred in cases with secondary displacement (7) and non-union (2). The frequency of these changes was higher in Jakob's type II fractures (p < 0.01), and it was associated with a greater initial displacement in both AP (p = 0.05) and lateral (p = 0.04) initial x-rays.

## Discussion

The primary objective of this research was to investigate non-displaced or minimally displaced LHCF in children and explore the associated complications. Efforts have been made to identify stable and unstable criteria for LHCF. An alternative approach is performing an arthrogram to assess the presence of a cartilaginous hinge, which could potentially indicate stability [5, 8, 9], but it requires conscious sedation. It can be helpful enhancing the reduction, but it may not be feasible for making the initial treatment at the emergency department. Additionally, interpreting the results of an arthrogram can be challenging [8, 14].

Other authors have tried to demonstrate that the displacement measured in the radiographs correlates with the existence of a cartilaginous hinge. Horn et al. defined a displacement ≤ 3 mm as the threshold to consider a fracture stable [8]. In our series, we were unable to find a relation between secondary displacement and the initial displacement of the fracture.

Although some authors recommend a non-operative treatment in all non-displaced [15], 24 out of our 72 patients who were non-operatively treated presented some complication. But, if we initially had treated them all surgically, we would have exposed a 54% of the patients to a harmful and unnecessary procedure. We found a 5% of avascular necrosis in the surgical group. It is worth noting that a surgery may adversely affect periostic vascularization, contributing to this complication, as well as an initial higher displacement.

Regarding the type of surgical treatment, closed reduction, and percutaneous pinning has been established as a reliable option for managing Milch II LHCF [16, 17]. In our study, we followed a specific sequence of action, where CRPP was the initial choice. Only when CRPP was not feasible, ORIF was initially elected. New meta-analysis and systematic reviews have shown no differences between these techniques [18–20].

We found 10% of the non-operatively treated fractures suffered a secondary displacement, most of them requiring a subsequent open surgery. Stratifying by type of fracture, 6% of Jakob’s I suffered an inadmissible displacement, as well as 17% of Jakob’s II. Similar percentages have been reported in the literature, ranging from 9.8 to 11.7% [12, 21–23]. None of surgically treated fractures experienced a secondary displacement. To early detect this complication, the general follow-up recommendation is to obtain an x-ray studio weekly for the first three weeks. However, other authors assumed that if subsequent displacement did not occur within the first week, it will never happen, so they do not recommend an x-ray until the third week [21]. No risk factors were found in our series, to help us in this decision process.

The incidence of non-union in our study was consistent with previous findings. We observed one case of non-union in each treatment group of Jakob’s II LCHF with a statistical significance (p = 0.05). These findings align with the literature published [21], indicating that Jakob's type II fractures may benefit from surgical intervention from the onset. Among the LHCF non-operatively treated, we observed a notably lower non-union rate of 1.4% in Jakob’s type I than 5.5% in Jakob’s type II. This rate is significantly lower than some previously published rate of 14.5% [12, 13], but in consonance with recent studies [17, 18, 24–26].

Some authors proposed surgical treatment for LHCF with a displacement higher than 1.6 mm [27]. Using ROC curve analysis, they reported an average area under the curve (AUC) of 0.75 and 0.79 for different authors. However, they did not differentiate between x-ray views in their evaluation. Our results indicate that surgical treatment of fractures with a displacement greater than 1.5 mm in either the AP or lateral views could effectively prevent non-union. Edmond et al. even stablished a 1.2-mm threshold to treat them surgically [28] and Li et al. asserted that the routine cutoff displacement of 2 mm may not reliably reflect the fracture stability as it was not specific whether the cartilage hinge was intact [29]. We observed a higher incidence of alignment disturbances when a displacement greater than 1.5 mm was present, with specificities of 72% in AP and 67% in lateral x-rays.

Our ROC analysis further revealed that a displacement greater than 1.5 mm in either the AP or lateral view has 100% sensitivity for predicting non-union. Since non-union often requires a more aggressive surgery, we propose that CRPP should be considered for fractures with a displacement exceeding 1.5 mm in the AP or lateral radiographs. Implementing this approach in our series would have meant operating on 31% of the patients. This approach would have included all cases with non-union and three out of the seven cases with secondary displacement. The differences in recommended thresholds highlight the ongoing debate and variability in the literature.

Hypertrophy of the lateral condyle emerged as the most common complication, with an incidence of 19%, being more frequent in patients surgically treated (50 versus 22%). However, it remains uncertain whether the hypertrophy is a result of the surgical approach, the higher initial displacement, or the normal process of healing, and even if it really is more an aesthetic problem. We observed a higher proportion of males than females in our study. Despite this disparity, we were unable to find the reason. None of these patients required further procedures with a completely functional elbow.

Our study has several limitations. It is a retrospective study, which limits the follow-up and the capability to collect data. A prospective study that follows patients until osseous maturity is attained, would provide more comprehensive information. Additionally, the sample size in our study is limited. Moreover, we were unable to obtain oblique x-ray exams in all our patients, which could have provided valuable information [30].

These findings provide valuable insights for determining the threshold for surgical intervention in LHCF cases. A displacement greater than 1.5 mm in either the AP or lateral view can be considered a point for considering surgical treatment to prevent some complications. The associations found in our study highlight the importance of future research to better understand treatment and outcomes of this children fracture.

## Data Availability

The datasets used and/or analysed during the current study are available from the corresponding author on reasonable request.

## Abbreviations

AP: Anteroposterior
AUC: Area Under the Curve
AVN: Avascular Necrosis
CRPP: Close Reduction Percutaneous Pinning
LHCF: Lateral Humeral Condyle Fracture
MRI: Magnetic Resonance Imaging
ORIF: Open Reduction Internal Fixation
ROC: Receiver Operating Characteristics
SD: Standard Deviation

## Declarations

### Strobe

Strengthening the Reporting of Observational Studies in Epidemiology (STROBE) guidelines have been followed.
